# Interactions between AMOT PPxY motifs and NEDD4L WW domains function in HIV-1 release

**DOI:** 10.1016/j.jbc.2021.100975

**Published:** 2021-07-17

**Authors:** Lara Rheinemann, Tuscan Thompson, Gaelle Mercenne, Elliott L. Paine, Francis C. Peterson, Brian F. Volkman, Steven L. Alam, Akram Alian, Wesley I. Sundquist

**Affiliations:** 1 Department of Biochemistry, University of Utah School of Medicine, Salt Lake City, Utah, USA; 2 Department of Biochemistry, Medical College of Wisconsin, Milwaukee, Wisconsin, USA

**Keywords:** HIV, AMOT, Nedd4L, WW domains, PPxY, AMOT, angiomotin, ESCRT, endosomal sorting complexes required for transport, FP, fluorescence polarization, HSQC, heteronuclear single-quantum correlation, RMSD, root mean square deviation, NOE, nuclear Overhauser effect, PPxY, Pro-Pro-x (any amino acid)-Tyr

## Abstract

Like most enveloped viruses, HIV must acquire a lipid membrane as it assembles and buds through the plasma membrane of infected cells to spread infection. Several sets of host cell machinery facilitate this process, including proteins of the endosomal sorting complexes required for transport pathway, which mediates the membrane fission reaction required to complete viral budding, as well as angiomotin (AMOT) and NEDD4L, which bind one another and promote virion membrane envelopment. AMOT and NEDD4L interact through the four NEDD4L WW domains and three different AMOT Pro-Pro-x (any amino acid)-Tyr (PPxY) motifs, but these interactions are not yet well defined. Here, we report that individual AMOT PPxY and NEDD4L WW domains interact with the following general affinity hierarchies: AMOT PPxY1>PPxY2>PPxY3 and NEDD4L WW3>WW2>WW1∼WW4. The unusually high-affinity of the AMOT PPxY1–NEDD4L WW3 interaction accounts for most of the AMOT–NEDD4L binding and is critical for stimulating HIV-1 release. Comparative structural, binding, and virological analyses reveal that complementary ionic and hydrophobic contacts on both sides of the WW–PPxY core interaction account for the unusually high affinity of the AMOT PPxY1–NEDD4L WW3 interaction. Taken together, our studies reveal how the first AMOT PPxY1 motif binds the third NEDD4L WW domain to stimulate HIV-1 viral envelopment and promote infectivity.

Like other enveloped viruses, the assembling HIV-1 virion must be wrapped in a host-derived membrane (termed envelopment) and the membrane must then be severed to release the infectious particle from the producer cell (termed budding). HIV-1 assembly is organized by the viral Gag protein (1, 2), and budding is mediated by the machinery of the cellular endosomal sorting complexes required for transport (ESCRT) pathway (3, 4). To initiate the budding process, PTAP and YPxL peptide motifs located within the C-terminal p6 region of Gag recruit two early-acting ESCRT factors, TSG101 (a component of the ESCRT-I complex) and ALIX, respectively. These factors then recruit downstream ESCRT complexes that constrict the viral bud neck and perform the membrane fission reaction that releases the virion from the host plasma membrane.

HIV-1 release also requires ubiquitin transfer (5, 6, 7). Precisely how ubiquitin promotes HIV-1 release is not yet fully understood, but other retroviral Gag proteins recruit members of the NEDD4 family of HECT-containing ubiquitin E3 ligases and promote budding (8). Recruitment of NEDD4 is typically facilitated by a PPxY motif (where P = proline, x = any amino acid, and Y = tyrosine). The PTAP, YPxL, and PPxY motifs are collectively termed “late domains” because their mutation arrests virion morphogenesis at late stages of assembly. Different late domains can act autonomously and interchangeably, implying that they likely perform analogous functions in recruiting ESCRT factors. In the case of PPxY–NEDD4 family E3 ligase interactions, this could occur through local ubiquitination of proteins at sites of virus assembly because at least three different ESCRT factors, TSG101/ESCRT-I, ALIX, and ESCRT-II, have ubiquitin-binding activities (8, 9, 10, 11, 12, 13, 14, 15).

Although HIV-1 Gag does not contain an identifiable PPxY late domain, the NEDD4 family member NEDD4L can nevertheless stimulate virion release (16, 17, 18). This stimulation is particularly pronounced when a naturally occurring isoform of NEDD4L, isoform 2 (also called NEDD4-2s or NEDD4L_ΔC2_), is overexpressed, and when TSG101/ESCRT-I and ALIX recruitment are impaired. HIV-1 constructs lacking the TSG101/ESCRT-I and ALIX recruiting late domains within p6^Gag^ (HIV- 1_ΔPTAP,ΔYP_) are therefore useful for assaying the NEDD4L elements and activities that stimulate virion release. Loss of endogenous NEDD4L also impairs HIV-1_ΔPTAP,ΔYP_ release, however, indicating that even at native levels, this enzyme can participate in a ubiquitin-dependent step of virus assembly (17).

We have also shown that the NEDD4L-binding partner angiomotin (AMOT) can associate with HIV-1 Gag and function together with NEDD4L to promote HIV-1 release (19). Importantly, AMOT is required for NEDD4L stimulation of HIV-1_ΔPTAP,ΔYP_ release, and the two proteins act synergistically. Stimulatory activity is specific to the p130 isoform of AMOT, whereas shorter isoforms such as AMOT p80 that cannot bind NEDD4L do not stimulate virion release. These observations imply that AMOT and NEDD4L function together during HIV-1 assembly and release. In the absence of AMOT, assembling wt virions fail to form fully spherical enveloped particles, indicating that AMOT acts upstream of the ESCRT machinery, during the envelopment phase of virion assembly (19). AMOT p130 contains a Bin/amphiphysin/Rvs-like domain that can remodel and tubulate membranes in other contexts (20, 21), suggesting that this domain could mediate AMOT p130 recruitment when the assembling virion reaches the proper stage of membrane curvature and/or help complete virion envelopment.

In addition to HIV-1, other enveloped viruses may utilize similar host complexes to help drive membrane envelopment and release. For example, another motin family member, angiomotin-like 1, links the structural M protein of paramyxoviruses with NEDD4 family members to promote virion release in an ESCRT- and ubiquitin-dependent pathway (22, 23). AMOT also enhances the release of filoviruses (24, 25), whose structural proteins contain PPxY late domains that recruit NEDD4 directly. Thus, these very different enveloped viruses appear to utilize similar egress machineries, and learning how the AMOT–NEDD4L complex functions in HIV-1 release will therefore likely have broader implications for understanding enveloped virus assembly.

Here, we have investigated how NEDD4L and AMOT interact with one another to stimulate HIV-1 release. The two proteins have been shown to bind one another through interactions that are mediated by PPxY motifs in AMOT and WW domains in NEDD4L (19, 26). PPxY–WW domain interactions mediate protein–protein interactions in many different cell signaling and trafficking pathways (27, 28), and their recognition modes and structures have been defined in many cases (27, 29, 30). Nevertheless, it is not yet clear precisely how AMOT and NEDD4L interact because the N-terminal region of AMOT contains three different PPxY motifs and the central region of NEDD4L contains four different WW domains (Fig. 1). We undertook this study with the goals of determining which of the 12 different possible pairwise AMOT PPxY–NEDD4L WW domain interactions are most energetically and functionally important and determining the structural basis for these more favorable interaction(s).

**Figure 1:**
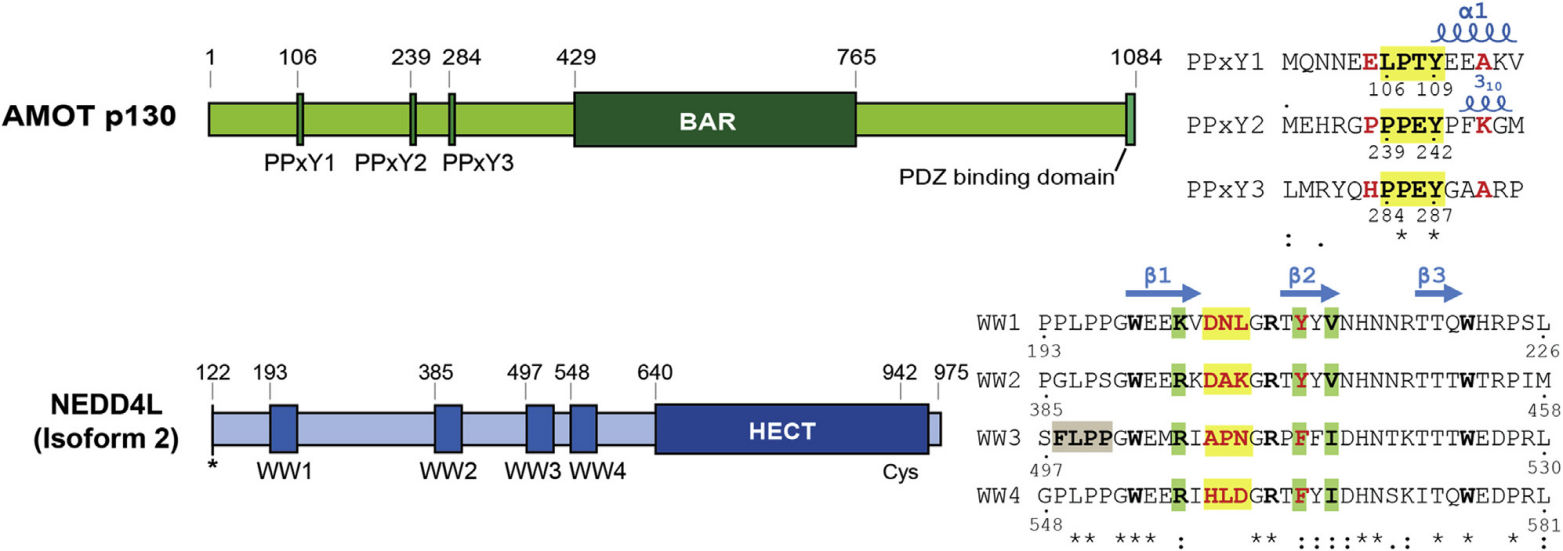
**Domain organization and motif sequences of AMOT p130 and NEDD4L.** PPxY color coding shows the core motif (*yellow highlight*), key variable residues (*red font*), and secondary structures (i + 4 α-helix in PPxY1 and i + 3 3_10_-helix in PPxY2). WW domain color coding shows the two eponymous Trp residues (*bold W*), loop 1 residues (*yellow highlights*), hydrophobic pocket residues (*green highlights*), key variable residues (*red*), and a cryptic motif that binds in the binding site of an adjacent subunit in the WW3 crystal lattice (*tan highlight*, Fig. S2). Positions of the three canonical β-strands are shown. Residue conservation in the PPxY and WW domain alignments is denoted with *asterisks* (full conservation), *colons* (strong conservation), and *dots* (weak conservation). Experiments were carried out using the naturally occurring isoform 2 of NEDD4L (also referred to as NEDD4-2s or NEDD4DC2). Isoform 2 lacks the 121 N-terminal residues of the C2 domain present in NEDD4L isoform 1 (*asterisk*). See Refs. (17, 18, 19) for a full explanation of NEDD4L isoforms and activities. PPxY, Pro-Pro-x (any amino acid)-Tyr.

## Results and discussion

### AMOT PPxY1 is required for efficient HIV-1 release and infectivity

**Figure 2:**
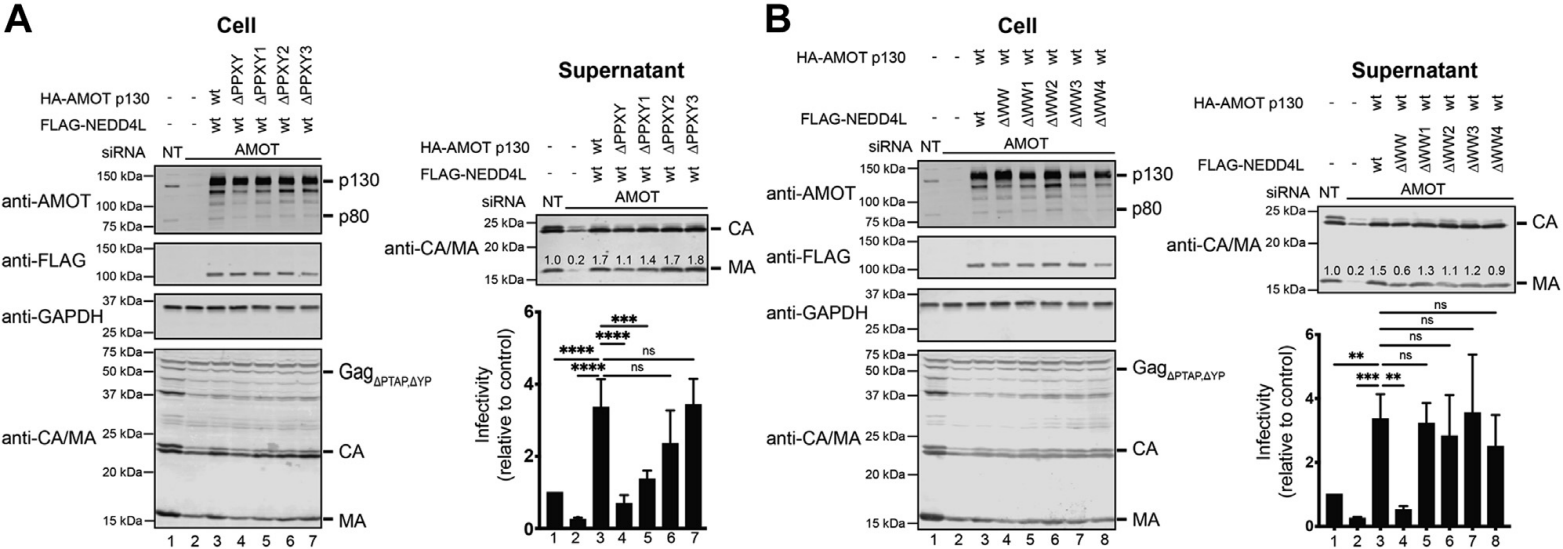
**Roles of AMOT–NEDD4L in HIV-1 release and infectivity.***A*, importance of AMOT PPxY motifs for NEDD4L-dependent release of HIV-1_ΔPTAP,ΔYP_. *Left panels* are Western blots showing HEK293T cellular levels of endogenous AMOT and exogenous HA-AMOT p130 or indicated mutants (panel 1, anti-AMOT), exogenous FLAG-NEDD4L (panel 2, anti-FLAG), endogenous GAPDH (panel 3, anti-GAPDH, loading control), and HIV-1 Gag_ΔPTAP,ΔYP_ and the MA and CA proteolytic processing products (panel 4, anti-MA and anti-CA). Cells were cotransfected with a nontargeting (NT) siRNA (lane 1) or an siRNA-targeting endogenous AMOT (lanes 2–7) and expression vectors for HIV-1_ΔPTAP,ΔYP,_ wt FLAG-NEDD4L (lanes 3–7), wt or mutant, siRNA-resistant HA-AMOT p130 (lanes 3–7), or an empty vector control (lanes 1 and 2). Right panels show corresponding levels of extracellular, virion-associated CA^Gag^ and MA^Gag^ proteins (panel 1, anti-MA and anti-CA) and viral titers (panel 2), relative to the value in the control experiment, set to 1.0. Titers for control experiment were 1–5 × 10^3^ infectious units per milliliter. Numbers within the blots show integrated intensities of the MA band (relative to the value in the control experiment, set to 1.0; average of three independent repeats). Here and in panel *B*, error bars denote SD from three independent replicates. ∗∗∗∗*p* < 0.0001, ∗∗∗*p* < 0.001, and ∗∗*p* < 0.01 by one-way ANOVA followed by Dunnett's multiple comparisons test. *B*, importance of WW domains for NEDD4L-dependent release of HIV-1_ΔPTAP,ΔYP_. Panels are equivalent to those in panel *A*, except that cells were cotransfected with a nontargeting (NT) siRNA (lane 1) or an siRNA-targeting endogenous AMOT (lanes 2–8), expression vectors for HIV- 1_ΔPTAP,ΔYP_ (lanes 1–8), a wt, siRNA-resistant HA-AMOT p130 construct (lanes 3–8), wt or mutant FLAG-NEDD4L (lanes 3–8), or an empty vector control (lanes 1 and 2). ns, not significant; PPxY, Pro-Pro-x (any amino acid)-Tyr.

We began by testing the functional requirements for each of the three AMOT PPxY motifs on release and infectivity, using an HIV-1_ΔPTAP,ΔYP_ construct that lacked functional PTAP and YPXL late domains and is therefore highly dependent on NEDD4L and AMOT (16, 17, 19). As expected (19), siRNA depletion of AMOT reduced HIV-1_ΔPTAP,ΔYP_ virion release into the supernatants of HEK293T producer cells, and correspondingly decreased viral titers, as measured in single-cycle infectivity assays (Fig. 2*A*, compare lanes 1 and 2). Conversely, virion release and infectivity were strongly stimulated by coexpression of wt NEDD4L and an siRNA-resistant AMOT p130 expression construct (lane 3), but not by an AMOT construct with AAxY mutations in all three PPxY motifs (ΔPPxY, lane 4). Mutations in each of the three individual PPxY motifs altered virus release and infectivity to different degrees. Specifically, mutation of PPxY1 strongly impaired virus release (ΔPPxY1, lane 5), whereas mutations in the second and third PPxY motifs had moderate (ΔPPxY2, lane 6) or no (ΔPPxY3, lane 7) effects. Thus, the AMOT PPxY motifs are required for efficient HIV- 1_ΔPTAP,ΔYP_ release and infectivity, and PPxY1 is the most important of the three motifs.

The importance of the individual NEDD4L WW domains was tested by coexpressing wt AMOT p130 together with wt and mutant NEDD4L constructs that carried inactivating Ala mutations in the essential second conserved Trp residue of each of the four different NEDD4L WW domains (Fig. 2*B*). A construct carrying mutations in all four WW domains failed to rescue HIV-1_ΔPTAP,ΔYP_ release and infectivity, confirming the importance of the PPxY–WW domain for virus release (ΔWW, lane 4). However, all four NEDD4L constructs with mutations in single WW domains (ΔWW1-4, lanes 5–8) stimulated virion release and infectivity to near wt levels, demonstrating that retention of three functional WW domains can compensate for the loss of any single other WW domain.

### AMOT PPxY1 binds with high affinity to the NEDD4L WW3 domain

**Figure 3:**
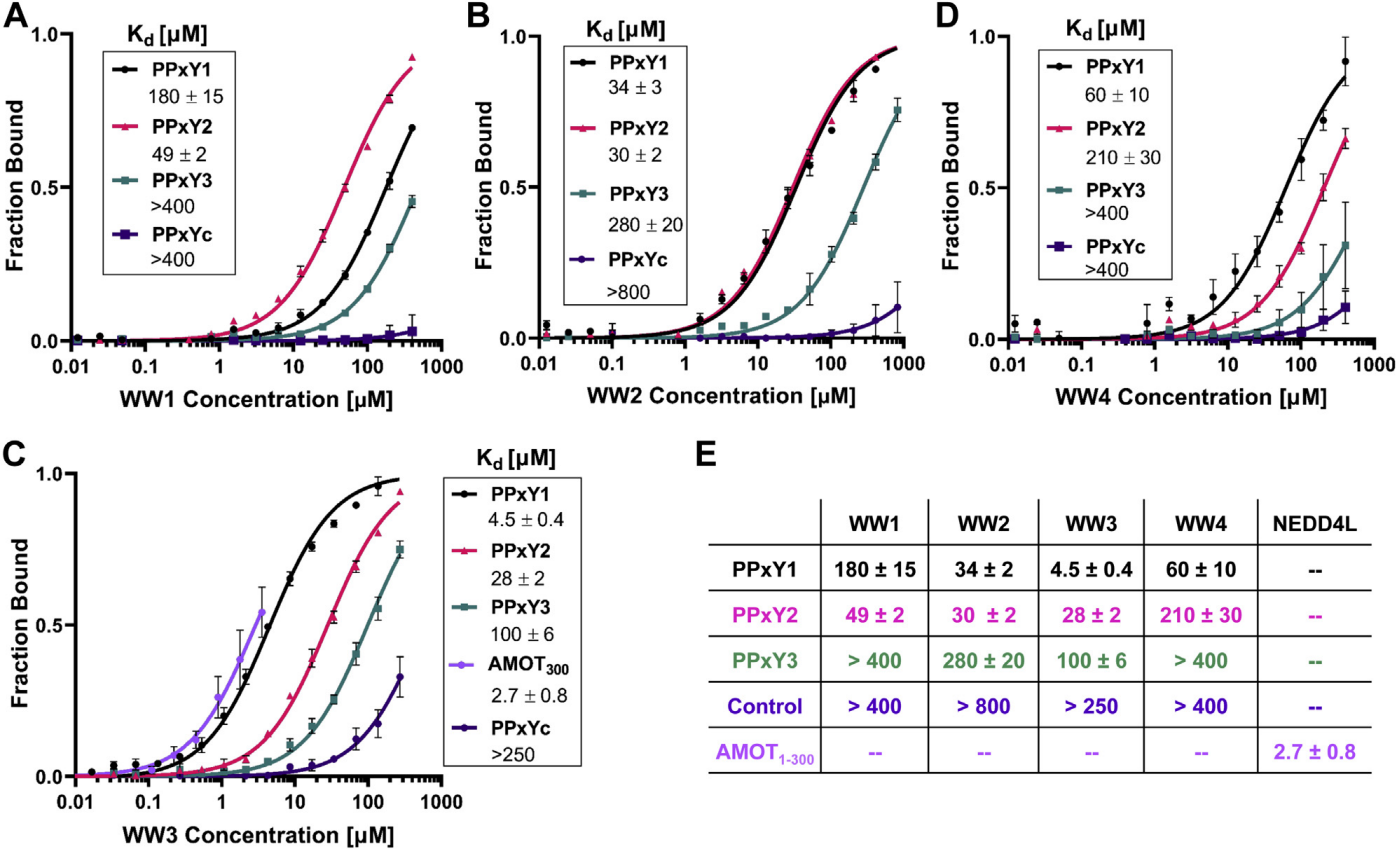
**Fluorescence polarization binding isotherms showing NEDD4L WW domains interacting with individual AMOT PPxY motifs**. *A*–*D*, binding isotherms of synthetic AMOT PPxY peptides to the four different recombinant NEDD4L WW domains. Binding was measured by changes in the fluorescence polarization (FP) of PPxY peptides with C-terminal Oregon Green fluorophores (pH 7.5 and 25 °C). Color coding: PPxY1 (*black*), PPxY2 (*pink*), PPxY3 (*green*), and the control peptide lacking a PPxY motif (*purple*). Note that the FP binding isotherm for AMOT_1-300_ and full-length NEDD4L (*violet*) is also shown in panel *C*. Data points report the mean of three independent titrations, and fitted curves follow the equation Y=(maxFP∗[protein]/(*K*_*d*_ + [protein])). Error bars denote SD. E, fitted *K*_*d*_ mean values (μM) ± mean SEM. PPxY, Pro-Pro-x (any amino acid)-Tyr.

We used fluorescence polarization (FP) anisotropy binding assays to determine the affinities of labeled peptides corresponding to each of the three AMOT PPxY motifs for each of the four different recombinant NEDD4L WW domains (Fig. 3, *A–D*). The 12 different pairwise interactions spanned more than two orders of magnitude in binding affinity (Fig. 3*E*), with the following trends: (1) NEDD4L WW3 was consistently the strongest PPxY binder, (2) NEDD4L WW4 was the weakest PPxY binder in two of three cases, and (3) AMOT PPxY3 was consistently the weakest WW domain binder. The AMOT PPxY1–NEDD4L WW3 interaction was of particular note because it was more than 5-fold tighter than any other pairwise interaction (*K*_*d*_ = 4.5 ± 0.4 μM).

In some contexts, tandem WW domains can bind cooperatively to tandem PPxY motifs, and this can even occur when one of the PPxY motifs is cryptic. An example is the tandem WW1/WW2 domains of the KIBRA protein, which form a well-ordered multidomain module that binds tightly (*K*_*d*_ = 96 nM) to AMOT PPxY3 (_284_PPEY_287_) and to a cryptic _278_LMRY_281_ motif located immediately upstream (31). To test whether the NEDD4–AMOT complex makes any such synergistic interactions, we quantified the binding of full-length recombinant NEDD4L to an Alexa Fluor–tagged recombinant AMOT construct that spanned all three PPxY motifs (AMOT_1-300_, Fig. 3, *C* and *E*). Although the limited solubility of full-length NEDD4L prevented sampling of the full isotherm, binding was observed with an apparent dissociation constant of 2.7 ± 0.8 μM. This interaction is only 1.5-fold tighter than that of the isolated AMOT PPxY1–NEDD4L WW3 complex, and we therefore conclude that this single interaction likely provides most of the binding energy in the AMOT_1-300_–NEDD4L complex. However, other PPxY–WW interactions also probably contribute to creating the slightly tighter binding observed in the AMOT_1-300_–NEDD4L complex, and we also cannot rule out the possibility of compensatory effects in the full-length protein. For example, autoinhibitory intramolecular WW domain interactions have been postulated to regulate NEDD4L and prevent self-ubiquitination (31). Nevertheless, our data indicate that the AMOT_1-300_–NEDD4L complex does not contain special high-affinity or highly cooperative interactions but rather that the different NEDD4L WW domains and AMOT PPxY motifs instead bind relatively independently, as has been seen in other cases where multiple WW domains can bind to multiple PPxY motifs (32, 33). This conclusion is consistent with the unique functional importance of the AMOT PPxY1 motif in HIV-1 release (Fig. 2*A*) and with a recent report that the homologous interaction also dominates binding of NEDD4 to angiomotin-like 1 (23).

To understand why the KIBRA WW1/WW2 element binds tightly to AMOT, whereas the similarly spaced, tandem NEDD4L WW3/WW4 element does not, we modeled NEDD4L WW3/WW4 based on the KIBRA WW1/WW2 structure (34) (Fig. S1). The model suggests that formation of a stable NEDD4L WW3/WW4 interdomain interface may be disfavored by the anionic Glu^526^ residue located between the WW3 and WW4 domains (replacing Ile^35^ in the equivalent position of KIBRA WW1/WW2). Consistent with this idea, the affinity of the KIBRA–AMOT complex is reduced 50-fold when Ile^35^ is mutated to Asp (34). Cooperative binding may also be disfavored by substitution of two other large hydrophobic KIBRA WW1/WW2 interface residues (Phe^47^ and Leu^57^) with smaller hydrophobics at equivalent positions in NEDD4L WW3/WW4 (Leu^542^ and Pro^552^, respectively).

### Structural studies of AMOT PPxY–NEDD4L WW complexes

Having established that the different NEDD4L WW domains and AMOT PPxY motifs bind independently, we next investigated the structural basis for the observed affinity differences in the different pairwise complexes. This was done by determining and comparing the structures of three different NEDD4L WW domains bound to AMOT PPxY motifs, including the high-affinity AMOT PPxY1–NEDD4L WW3 complex.

**Figure 4:**
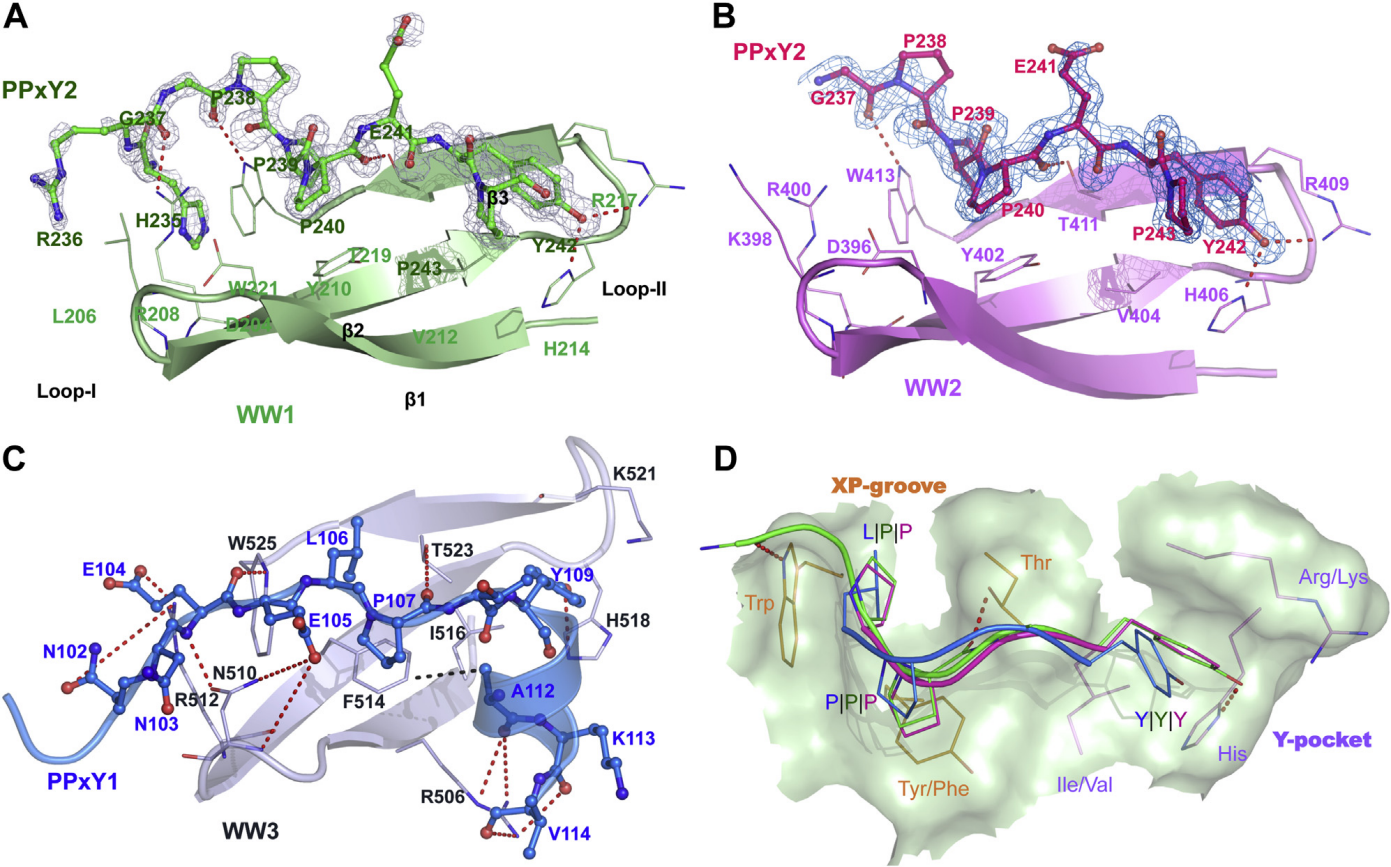
**Structures of AMOT PPxY–NEDD4L WW complexes**. *A*, crystal structure of AMOT PPxY2 (*green sticks*, chain b) bound to NEDD4L WW1 (*pale green*, chain a; *ribbon diagram* with key residue side chains shown explicitly). The three WW domain strands are labeled in this panel. *B*, crystal structure of AMOT PPxY2 (*magenta*, chain d) bound to NEDD4L WW2 (*pink*, chain c). *Red dashed lines* in panels *A* and *B* denote observed hydrogen bonds. Mesh renderings show the F_O_-F_C_ map (3σ), calculated with the PPxY peptide omitted. *C*, solution structure of AMOT PPxY3 (*blue*) bound to NEDD4L WW3 (*gray*). *Dashed red lines* indicate observed hydrogen bonds or salt bridge interactions. The hydrophobic pocket interaction between WW3 Phe^514^ and PPxY1 Ala^112^ (∼3.9 Å) is shown as *black dashed lines*. Glu^105^ interacts with Asn^510^ (2.7–4.5 Å) in ∼50% of ensemble structures. Each of the residues Glu^104^ and Asn^102^ individually interacts with Arg^512^ (2.6–4.5 Å) in ∼40% of ensemble structures. Carbonyl oxygen of Glu^105^ hydrogen bonds to side-chain nitrogen of Asn^510^ (not shown for clarity). Arg^506^ interacts with the carbonyls of Ala^112^, Lys^113^, and Val^114^ (3.0–4.5 Å) in ∼25% of ensemble structures. *D*, surface rendering of the conserved WW core domain showing the characteristic XP groove (residue labels in *orange*) and Y-pocket (residue labels in *purple*). PPxY peptides are overlaid and colored following panels *A-C*. *Red dashed lines* denote canonical WW–PPxY hydrogen bonds (shown only for WW1–PPxY2 for clarity). PPxY, Pro-Pro-x (any amino acid)-Tyr.

To obtain structures, we screened a series of different high- and moderate-affinity AMOT PPxY–NEDD4L WW complexes for crystallization. Two such complexes, AMOT PPxY2–NEDD4L WW1 and AMOT PPxY2–NEDD4L WW2, yielded high-resolution crystal structures (1.52 Å and 1.74 Å, respectively) (Table S1 and Fig. 4, *A* and *B*).

Attempts to crystallize the high-affinity AMOT PPxY1–NEDD4L WW3 complex repeatedly yielded crystals of NEDD4L WW3 alone. The explanation for this became evident when we determined the apo-NEDD4L WW3 structure and observed that in the preferred crystal lattice, a proline-rich element from the N terminus of one NEDD4L WW3 domain (cryptic peptide, residues _498_FLPP_501_, Fig. 1) bound in the PPxY-binding site of a neighboring molecule (Fig. S2*A*). The cryptic peptide binds in the opposite orientation of AMOT PPxY peptides (Fig. S2*B*) and is therefore not discussed further.

In the absence of suitable crystals, we used NMR spectroscopy to determine the solution structure of the AMOT PPxY1–NEDD4L WW3 complex. To drive complex formation and simplify NMR assignment/refinement strategies, we determined the structure of a recombinant construct in which the AMOT PPxY1 motif was fused to the NEDD4L WW3 N terminus through a short Gly–Ser linker. The intramolecular PPxY1 element bound in a native conformation within this construct, as evidenced by the nearly perfect overlap of ^1^H/^15^N heteronuclear single-quantum correlation (HSQC) signals for WW3 residues of the fusion construct and a nonfused NEDD4L WW3 titrated with AMOT PPxY1 peptide (Fig. S3, *A* and *B*). NMR spectra of the PPxY1-WW3 fusion protein were of high quality, and the resulting structure (Fig. 4*C*) was well ordered for the residues corresponding to AMOT residues _101_QNNEELPTYEEAKV_114_ and NEDD4L WW3 residues 498 to 526 (0.5 Å root mean square deviation (RMSD) for 20 lowest energy structures) (Fig. S3*C* and Table S2). Finally, to identify conformational changes that occur upon AMOT PPxY1 binding, we also determined a high-resolution NMR solution structure of the unliganded NEDD4L WW3 protein (Table S2).

All five of our new structures exhibit canonical WW domains, with three-stranded β-sheets and canonical positioning of the two eponymous Trp residues (27, 29, 30). The three WW–PPxY complexes and the WW3 cryptic peptide complex are superimposable (<1.0 Å RMSD), whereas the apo WW3 WW domain structure (∼3.0 Å RMSD) differed in significant ways, as discussed below.

In all three WW–PPxY complexes, the PPxY core elements bind in an extended polyproline II helical conformation across the concave WW sheet (Fig. 4, *A–C*). As illustrated in Figure 4*D*, each conserved residue in the PPxY motif makes canonical binding interactions: the first Pro/Leu residue binds in the “XP-groove,” where it stacks against the second conserved WW Trp residue (Trp^525^ in WW3). The indole nitrogen of this Trp residue also hydrogen bonds to the backbone carbonyl located two residues before the PPxY motif. The second PPxY Pro packs against a Tyr/Phe residue from the second β-strand (β2), and its carbonyl oxygen forms a hydrogen bond to the hydroxyl of a conserved Thr (in β3). The fourth Tyr residue of PPxY binds in the characteristic “Y-pocket”, forming a hydrogen bond with conserved His (in loop-II between β2 and β3). Thus, the core WW–PPxY interactions are very similar in every case and apparently do not explain why AMOT PPxY1 binds WW3 with unusually high affinity.

### The unique Leu106 residue in AMOT PPxY1

**Figure 5:**
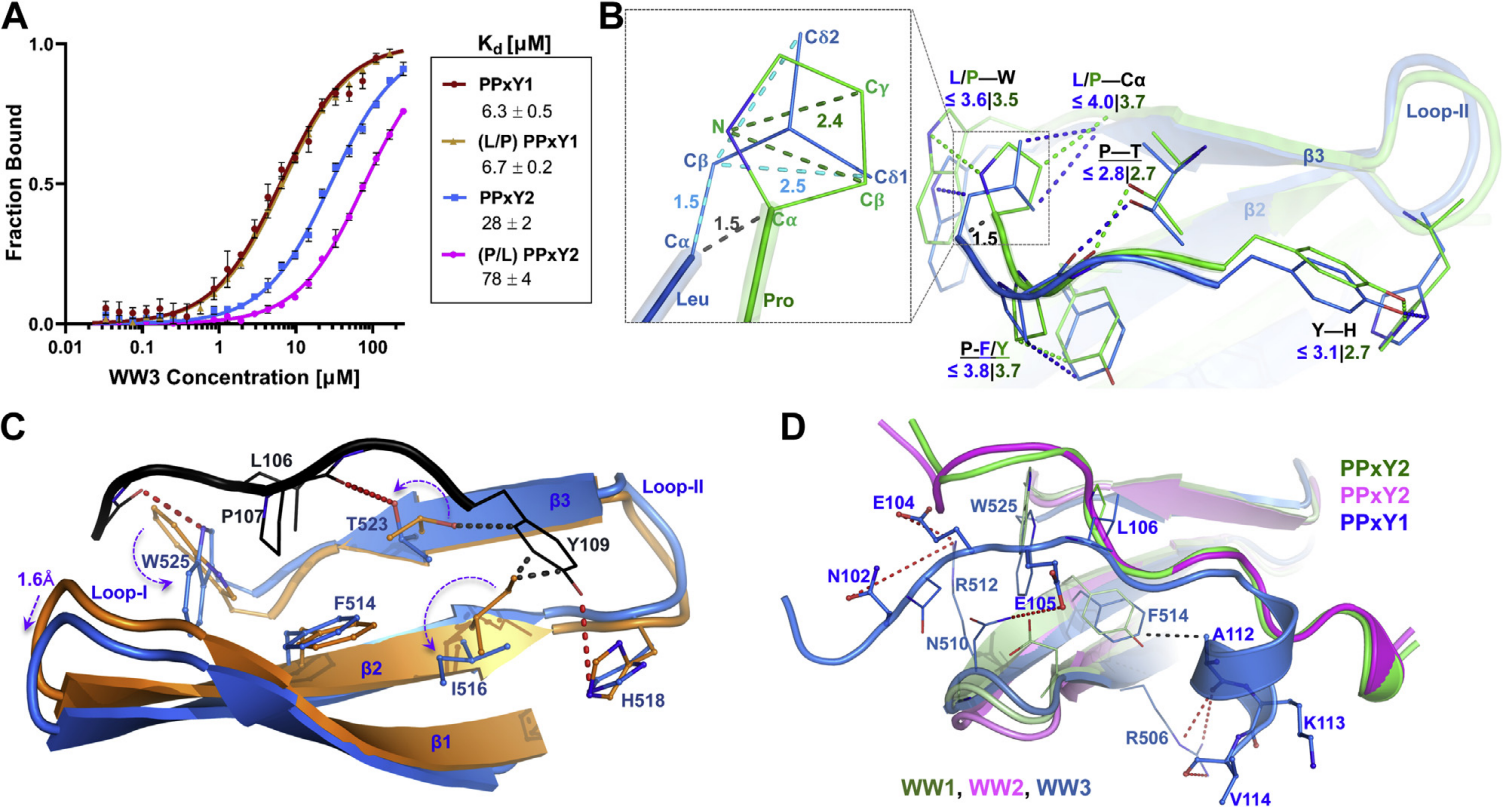
**Energetic and structural consequences of NEDD4L WW3 binding to wt and mutant AMOT PPxY motifs.***A*, fluorescence polarization (FP) binding isotherms and fitted *K*_*d*_ values (μM) for NEDD4L WW3 binding to peptides corresponding to wt AMOT PPxY1 (*red*), an PPxY1 L106P mutant (*orange*, denoted (L/P)PPxY1), wt PPxY2 (*blue*), and a PPxY2 P239L mutant (*magenta*, denoted (P/L)PPxY2). Error bars denote SD from four independent replicates. *B*, superpositions of PPxY1–WW3 (*blue*) and PPxY2–WW1 (*green*) complexes illustrating how the noncanonical Leu^106^ residue of the PPxY1 occupies the same position as the canonical Pro^239^ of PPxY2. Key conserved residues are shown explicitly; AMOT PPxY–WW distances (*dashed lines*, Å) are shown. WW3–PPxY1 distances are from ∼75% of ensemble structures. Inset: close-up view of Leu/Pro residues within the *boxed* region. Note that the Pro pyrrolidine ring and the Leu isopropyl group occupy the same WW binding site (Trp, Thr, Tyr/Phe, and His) and bury similar accessible surface areas (∼90 Å^2^). Distances (Å, *dashed lines*) are shown for scale and to illustrate the 1.5 Å shift between the PPxY1 Leu^106^ and PPxY2 Pro^239^ Cα positions. Substitution of Leu for Pro in the first position of the PPxY motif should reduce the propensity to form a PPII helix (37), but this unfavorable change may be compensated by burial of the larger Leu side chain. *C*, superposition of WW3 structures in the apo/unliganded (*orange*) and PPxY1 (*blue*) states. Displacements of the loop 1 and key side chains upon peptide (*black*) binding are shown as *purple arrows* and *dashed lines*. *Red dashed lines* depict canonical hydrogen bonds, and *black dashed lines* show steric clashes that require WW3 Ile^516^ (1.5 Å to Tyr^109^) and Thr^523^ (3.0 Å to Tyr^109^) to change conformation. *D*, superposition of PPxY2–WW1 (*green*), PPxY2–WW2 (*magenta*), and PPxY1–WW3 (*blue*), emphasizing the interactions unique to WW3–PPxY1. PPII, polyproline II; PPxY, Pro-Pro-x (any amino acid)-Tyr.

**Figure 6:**
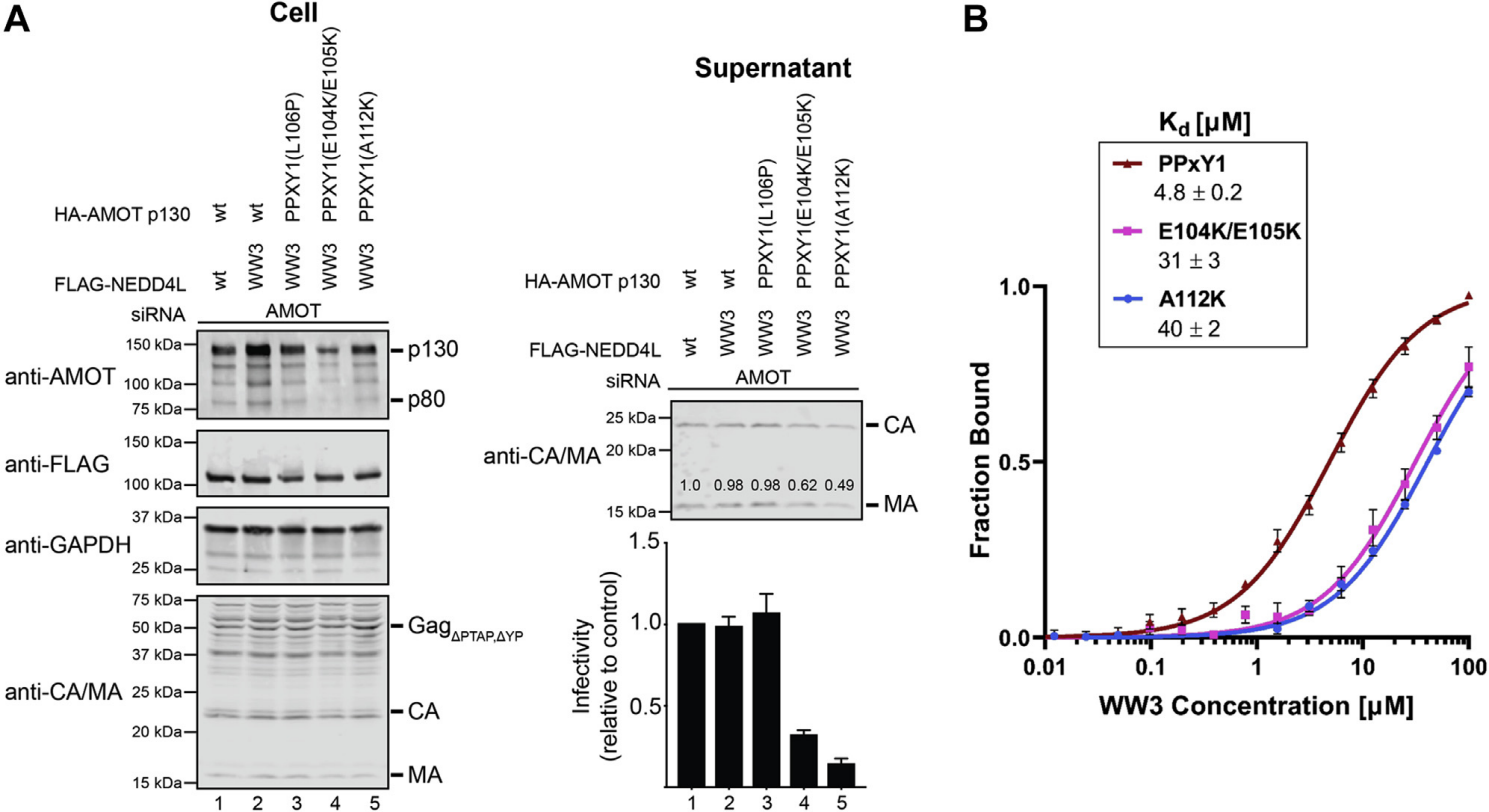
**Impact of unique PPxY1 N- and C-terminal extensions on NEDD4L WW3 binding and HIV budding and infectivity.***A*, importance of AMOT PPxY1 residues for NEDD4L_WW3_-dependent release of HIV-1_ΔPTAP,ΔYP_. Left panels are Western blots showing HEK293T cellular levels of endogenous AMOT and exogenous HA-AMOT p130 or indicated mutants (panel 1, anti-AMOT), exogenous FLAG-NEDD4L (panel 2, anti-FLAG) or FLAG- NEDD4L_WW3_, endogenous GAPDH (panel 3, anti-GAPDH, loading control), and HIV-1 Gag_ΔPTAP,ΔYP_ and the MA and CA proteolytic processing products (panel 4, anti-MA and anti-CA). Cells were cotransfected with an siRNA-targeting endogenous AMOT and expression vectors for HIV-1_ΔPTAP,ΔYP_, wt FLAG-NEDD4L (lane 1), FLAG-NEDD4L_WW3_ (lanes 2–5), and wt or mutant, siRNA-resistant HA-AMOT p130. Right panels show corresponding levels of extracellular, virion-associated CA^Gag^ and MA^Gag^ proteins (panel 1, anti-MA and anti-CA) and viral titers (panel 2), relative to the value in lane 1, set to 1.0. Numbers within the blots show integrated intensities of the MA band intensities (relative to the value in the control experiment, set to 1.0; average of two independent repeats). Error bars denote the SD from two independent replicates. *B*, importance of AMOT PPxY1 residues for NEDD4L_WW3_ binding. Fluorescence polarization binding isotherms and *K*_*d*_ values (μM) for the interaction of WW3 with wt PPxY1 (*red*), and the E104K/E105K (*pink*) and A112K (*blue*) mutants. Error bars denote the SD from three independent replicates. PPxY, Pro-Pro-x (any amino acid)-Tyr.

The AMOT PPxY1 motif is unique in having a Leu^106^ residue in place of the canonical Pro in the first position (Fig. 1). We therefore investigated whether this residue might underlie the uniquely high affinity of the PPxY1–WW3 interaction. This was not the case, however, because substitution of Leu^106^ by Pro did not measurably alter NEDD4L WW3 binding affinity (Fig. 5*A*). Similarly, mutation of Leu^106^ to Pro did not alter the ability of AMOT to stimulate HIV_ΔPTAP,ΔYP_ release and infectivity to the same extent as NEDD4L wt (Fig. 6*A*, compare lanes 2 and 3). These experiments were performed in the context of NEDD4L constructs in which the WW1, WW2, and WW4 elements were inactivated by mutation and only the WW3 domain was active (denoted NEDD4L_WW3_). These experiments showed that the NEDD4L_WW3_ construct alone could rescue HIV release and infectivity to wt levels (Fig. 6*A*, compare lanes 1 and 2), and this construct was used to ensure that any effects observed were restricted to the PPxY1–WW3 interaction. Thus, the unique Leu^106^ residue does not play a key binding or functional role although it could, in principle, contribute to specificity by helping to discriminate between different WW domains in NEDD4L and other WW proteins.

Our structural analyses reveal that the isopropyl Cβ-Cδ element of AMOT PPxY1 Leu^106^ occupies the same WW pocket as the pyrrolidine ring of the canonical first Pro residue (*e.g.*, Pro^239^ in PPxY2, Fig. 5*B* inset). To accommodate the longer Leu side chain, the PPxY1 backbone shifts back by ∼1.5 Å as compared with PPxY2 backbone. We found, however, that equivalent Pro to Leu substitutions were not isoenergetic in all PPxY motifs, because substitution of Pro^239^ to Leu reduced PPxY2 binding to WW3 by 2.8-fold (Fig. 5*A*).

### Conformational changes that accompany AMOT PPxY1 binding to NEDD4L WW3

Comparison of the peptide-bound and -unbound AMOT WW3 structures revealed that peptide engagement expands the central binding groove and creates the different subpockets that accommodate conserved PPxY residues (Fig. 5*C*). The groove slightly expands (<2.0 Å) by the movement of loop I away from loop II/β3. Five WW domain sidechains also rotate significantly: Trp^525^ rotates to stack against PPxY1 Leu^106^ and to align the indole nitrogen to hydrogen bond to a carbonyl upstream of PPxY motif; His^518^ rotates to stack and hydrogen bond with the PPxY1 Tyr^109^ hydroxyl; Phe^514^ slightly moves upward to stack against PPxY1 Pro^107^; Ile^516^ and Thr^523^ rotate to accommodate the stacking of PPxY1 Tyr^109^ in the Y-pocket and align the Thr^523^ hydroxyl to hydrogen bond with the PPxY1-Pro^107^ carbonyl (Fig. 5*C*). Analogous “induced fit” peptide-binding mechanisms have been described for other WW–PPxY interactions (35).

### Structural basis for the high-affinity PPxY1–WW3 interaction

Comparison of the structure of PPxY1–WW3 to the lower affinity PPxY2–WW1 and PPxY2–WW2 complexes revealed that the high-affinity binding of PPxY1 to WW3 reflects a series of favorable contacts made uniquely in this complex by the N- and C-terminal extensions that flank the conserved PPxY core (Figs. 4*C* and 5*D*). In contrast, the PPxY2–WW1 and PPxY2–WW2 complexes make few favorable contacts outside the core (Figs. 4, *A* and *B* and 5*D*).

On the N-terminal side, the PPxY1 backbone approaches WW3 loop-I more closely (∼6 Å) than PPxY2 in the WW1 or WW2 complexes (11 Å and 14 Å, respectively) (Fig. 5*D*). Favorable side-chain interactions include hydrogen bonds between the PPxY1 Glu^104^ backbone nitrogen with the unique WW3 Asn^510^ side chain (a Leu in WW1 and Lys in WW2), and PPxY1 Glu^105^ Oε with the WW3 Asn^510^ backbone and side-chain nitrogen. The anionic and hydrophilic side chains of PPxY1 Asn^102^ and Glu^104^ also interact favorably with the WW3 Arg^512^ guanidinium (Figs. 4*C* and S4).

To assess the importance of the N-terminal WW3–PPxY1 interaction, we mutated AMOT PPxY1 Glu^104^ and Glu^105^ to Lys. This mutation reduced the affinity of the WW3–PPxY1 interaction 6-fold (from 4.8 to 31 μM) (Fig. 6*B*) and also reduced the ability of AMOT to stimulate virus release and infectivity in concert with the NEDD4L_WW3_ construct (Fig. 6*A*, compare lanes 2 and 4). Thus, the WW3 loop I composition contributes to PPxY1 binding, in good agreement with other studies (32, 33, 36, 37).

On the C-terminal side of the core, PPxY1 residues _109_YEEAKV_114_ form a short α-helix, capped by Thr^108^, that sits in a hydrophobic groove created by WW3 Phe^514^, Ile^516^ and the aliphatic atoms of Lys^521^ and Thr^523^ (Figs. 4*C* and 5*D*) Two complementary residues enable this interaction: the aromatic WW3 Phe^514^ side chain (as opposed to the larger, hydroxylated Tyr residue in WW1/WW2) and a matching small PPxY1 α-helix packing interface residue, Ala^112^, that fits well in the hydrophobic pocket (as opposed to a larger Lys residue in the same position in the 3_10_-helix of PPxY2). Tight PPxY1–WW3 packing also allows the conserved WW3 Arg^506^ residue to interact with the carbonyl oxygens of PPxY1 Ala^112^, Lys^113^, and Val^114^ (Fig. 4*C*). Analogous helix–hydrophobic groove interactions have also been observed in other PPxY–WW domain complexes (36, 37, 38).

To test the energetic contribution of the C-terminal PPxY1 helix–WW3 interaction, we mutated AMOT PPxY1 Ala^112^ to Lys. This mutation reduced WW3 binding affinity 8-fold (from 4.8 to 40 μM) (Fig. 6*B*). This mutation also reduced the ability of AMOT to stimulate virus release and infectivity in concert with the NEDD4L_WW3_ construct (Fig. 6*A*, compare lanes 2 and 5). Thus, the AMOT PPxY1 Ala^112^ residue contributes to the structure, affinity, and function of WW3 binding.

### Summary

The cellular host proteins AMOT and NEDD4L can cooperate to stimulate HIV-1 release, particularly when ESCRT factor recruitment is inefficient (19). The two proteins bind one another through interactions between the three PPxY motifs in AMOT and the four WW domains in NEDD4L, and these interactions are essential for AMOT- and NEDD4L-dependent HIV-1 release and infectivity (Fig. 2). We find that the AMOT PPxY1 element plays an essential role in this process and binds the NEDD4L WW3 domain with unusually high affinity (Figs. 2 and 3). This affinity reflects mutual compatibility between the unique PPxY1 N- and C-terminal extensions and the complementary WW3 Loop-I and Phe^514^/Ile^516^/Arg^506^ hydrophobic pocket elements, respectively (Figs. 4*C* and 5*D*). Sequence variations prevent all other possible AMOT PPxY–NEDD4L WW combinations from making this full set of interactions, thereby explaining the uniquely favorable affinity and specificity of the PPxY1–WW3 interaction. In the context of an HIV infection, the AMOT–NEDD4L interaction helps drive virion envelopment (19). We speculate that AMOT also helps recruit NEDD4L ubiquitin ligase activity to the nascent virion at the proper time. This could occur if the AMOT Bin/amphiphysin/Rvs domain senses the extent of virus assembly by assessing the degree of membrane curvature. Once the proper degree of membrane curvature is sensed, AMOT might then recruit and activate NEDD4L, thereby promoting recruitment of the early-acting, ubiquitin-binding ESCRT factors required to initiate bud neck closure and membrane fission.

## Experimental procedures

### Plasmids

Constructs for expressing wt and mutant NEDD4L and AMOT proteins in *Escherichia coli* and human HEK293T cells were created by standard cloning and mutagenesis methods, with detailed methods available upon request. Expression constructs are described in detail in Table S3. All of the new expression constructs used in our studies have been submitted to the Addgene plasmid repository (https://www.addgene.org/)

### Cell culture

HEK293T and HeLa-TZM-bl cells were maintained in Dulbecco's modified Eagle's medium (DMEM) (Thermo Fisher Scientific) containing 10% fetal bovine serum at 37 °C and 5% CO_2_. HEK293T cells were obtained from the American Type Culture Collection, and HeLa–TZM-bl cells were obtained from the HIV Reagent Program. Cells were tested for *Mycoplasma* contamination every 3 months using a PCR *Mycoplasma* Detection Kit (Applied Biological Materials).

## Budding assays

HIV-1 budding assays were performed as follows: HEK293T cells (3 × 10^5^ cells/well, 6-well plate) were transfected with 20 nM siRNA-targeting AMOT (19) using 7.5-μl Lipofectamine RNAiMax (Thermo Fisher Scientific). 24 h later, the medium was changed and cells were cotransfected with 20 nM siRNA-targeting AMOT, 0.5-μg HIV-1_NL4-3_ R9_ΔPTAP,ΔYP_, 0.5 μg of pCI-FLAG-NEDD4L, or indicated NEDD4L mutants and 1-μg pcDNA3 HA–AMOT p130 or indicated AMOT mutants. The medium was changed to fresh DMEM 6 to 8 h after transfection. Forty-eight hours after the second transfection, cells were harvested for Western blot analyses and supernatants harvested for titer measurements and virus purification.

Cells were washed off the plate in 1 ml PBS, pelleted by centrifugation, and lysed for 15 min at 4 °C in 150-μl RIPA buffer (10 mM Tris, 1 mM EDTA, 0.5 mM EGTA, 1% Triton X-100, 0.1% sodium deoxycholate, 0.1% SDS, and 140 mM NaCl, pH 8.0) supplemented with mammalian protease inhibitor (Sigma-Aldrich). Insoluble material was removed by centrifugation (10 min, 15,000*g*, 4 °C). 150 μl of 2 × Laemmli SDS-PAGE loading buffer supplemented with 10% 2-mercaptoethanol (Sigma-Aldrich) were added and samples were boiled for 5 min. Virions from 1-ml supernatant were pelleted by centrifugation through a 200-μl 20% sucrose cushion (90 min, 15,000*g*, 4 °C) and denatured by adding 100 μl 1× Laemmli SDS-PAGE loading buffer and boiling for 5 min. Proteins were separated by SDS-PAGE, transferred onto PVDF membranes, and probed with antibodies. Primary antibodies were as follows: anti-AMOT (1;3000, UT691, Covance), anti-FLAG (1:10,000, F1804, Sigma-Aldrich), anti-HIV CA (1:1000, UT415, Covance), anti-HIV MA (1:1000, UT556, Covance), and anti-GAPDH (1:10,000, MAB374, EMD Millipore, Burlington, VT). Antibodies with UT designations and numbers were raised against recombinant proteins purified in the Sundquist laboratory. Bands were visualized by probing the membrane with fluorescently labeled secondary antibodies (LI-COR Biosciences) and scanning with an Odyssey Imager (LI-COR Biosciences).

HIV-1 titers were assayed in HeLa-TZM-bl indicator cells using a β-galactosidase assay. HeLa-TZM-bl cells (5000 cells per well, 96-well plate) were infected with three different dilutions of virus-containing culture media, each in triplicate, harvested after 48 h, washed once with 200-μl PBS, and lysed in 50-μl 25 mM Tris phosphate (pH 7.8), 2 mM DTT, 2 mM 1,2-diaminocyclohexane N,N,N′,N′-tetraacetic acid, 10% glycerol, and 1% Triton X-100 (10 min, 23 °C). Cell lysates were incubated with 50-μl assay buffer (200 mM sodium phosphate (pH 7.3), 2 mM MgCl_2_, 100 mM β-mercaptoethanol, 1.33 mg/ml ortho-nitrophenyl-β-galactoside) for 60 min at 37 °C. Reactions were terminated by addition of 200-μl 1 M Tris base, and absorbance at 420 nm was read in a plate reader.

### Protein expression, purification, and labeling

Bacterial expression plasmids containing either GST-PP-WW or His-SUMO-WW domains, His-AMOT(1–300), or His-sortase (*Staphylococcus aureus*) were expressed in the Rosetta-pLysS bacterial strain grown in autoinduction media ZYP-5052 (39). Lysis buffer contained 50 mM Tris, 150 to 500 mM NaCl, 5% glycerol, 2 mM MgCl_2_, 1 mM TCEP, 0.5% NP-40, pH 7.5, supplemented with Roche cOmplete EDTA-free protease inhibitors, and 5 mM imidozole for His-tagged proteins. Lysate viscosity was reduced by addition of DNase (12.5 μg/ml, Roche) followed by ultrasonic disruption. Proteins captured on Ni-NTA cOmplete resin (Roche), or Glutathione Superose resin (GE Healthcare Bio-Sciences), were washed with the lysis buffer and eluted with the lysis buffer supplemented with 250 mM imidazole for His-tagged proteins, or GST-fusion proteins were separated from the GST tag by on-column cleavage with PreScission protease (1 nmol protease/30 nmol purified protein, GE Healthcare Bio-Sciences). The His-SUMO-Fusion proteins were further cleaved with ULP1 (1 nmol protease/30 nmol purified substrate protein). Further ion-exchange (pH 7.5, SP-column, HiTrap HP-SP, GE Healthcare Bio-Sciences) and S75 sizing column (150 mM NaCl, pH 7.5) purifications were performed, and the samples were snap-frozen for storage at −80 °C. Typical yields per liter culture were 60 nmol for WW domains, and 8 nmol for sortase and AMOT_1-300_. Protein identities were confirmed by electrospray mass spectrometry, which confirmed the expected full protein masses.

A labeled peptide (GGG-ALEXA488) was added to AMOT_1-300_ (which carried a sortase tag) by transpeptidation with sortase. Typical reactions contained 10 to 50 μM AMOT_1-300_, 30 to 150 μM GGG-ALEXA488 peptide and 20 μM sortase enzyme in the reaction buffer (50 mM Tris [pH 7.5], 150 mM NaCl and 10 mM CaCl_2_). Reactions were allowed to proceed at either room temperature or 4 °C until the reaction was complete (typically 1–4 h). AMOT_1-300_–ALEXA488 was then purified by ion-exchange chromatography (SP HiTrap) as described above.

### FP binding experiments

#### Peptide preparation

Three AMOT PPxY peptides (residues 100–114, 233–247, and 278–292, see Fig. 1) and one AMOT control peptide (residues 196–209), each containing a non-native, N-terminal cysteine, were synthesized, purified, and fluorescently labeled in the University of Utah Peptide Synthesis Core.

Briefly, peptides were synthesized with a cysteine at the N terminus using Fmoc solid-phase technology, common protecting groups, and HBTU chemistry on an ABI 433 synthesizer (Applied Biosystems). Synthesized peptides were purified by reversed-phase chromatography using acetonitrile/water gradients with 0.1% TFA in both solvents. Peptide-containing fractions were pooled, dried, and redissolved in DMSO. Fluorescent labeling was performed in DMSO at 4 °C with approximately 1.3-fold molar excess of Oregon Green 488 maleimide (Life Technologies/Molecular Probes 06034) dissolved in a 1:1 solution of acetonitrile:DMSO. Reaction progress was monitored by HPLC, and labeled peptides were separated from free dye and residual unlabeled peptides using the same reversed-phase conditions described above. Labeled peptide masses were determined by MALDI-TOF-MS at the University of Utah Mass Spectrometry Core facility: dye-labeled PPxY1 was 2358.9 Da (theoretical 2360.5 Da); dye-labeled PPxY2 was 2336.9 Da (theoretical 2338.6 Da); dye-labeled PPxY3 was 2350.0 Da (theoretical 2351.6 Da); and dye-labeled PPxY-control was 2090.8 Da (theoretical 2092.2 Da). Confirmed peptide fractions were dried under vacuum and redissolved and neutralized in water. Concentrations were calculated using the absorbance of Oregon Green 488 at 491 nm (extinction coefficient 83,000 cm^−1^ M^−1^ in 50 mM potassium phosphate, pH 9.0) before diluting in FP binding buffer (see below).

#### FP binding assay

FP experiments were performed in 100-μl reaction volumes in the binding buffer (20 mM Tris [pH 7.5], 150 mM NaCl, 0.5 mM TCEP) using 1 nM fluor-labeled PPxY peptides and 2-fold dilutions of WW domains (or associated mutants; WW1: 4 × 10^2^–4.8 × 10^−5^ μM; WW2: 8.2 × 10^2^−9.8 × 10^−5^ μM; WW3: 1.9 × 10^2^–2.3 × 10^−5^ μM; WW4: 4 × 10^2^–4.8 × 10^−5^ μM). Each dilution series was performed at least in triplicate. Each dilution (70 μl) was placed in a Corning 384 flat-bottom plate and allowed to equilibrate (26 °C, 15 min) in the plate reader. Measurements were taken in the BioTek Synergy Neo Hybrid Multi-Mode Microplate Reader. The instrument measured 50 data points for each well, with a 200-millisecond delay between wells. The lamp was positioned 9.0 mm away from the plate with excitation at 498 nm and emission monitored at 528 nm. The instrument gain was set on the control peptide sample, which consistently had a lower fluorescent intensity than the other peptides. Each binding isotherm was measured at least four independent times, with a one-minute delay between each read. Data were exported into Microsoft Excel and input into Graph Prism-6 (GraphPad, San Diego, CA). Curve fitting and *K*_*d*_ values were created using a pre-existing protocol in Graph Prism 6. The data were fit to the equation Y = (maxFP∗[protein])/(*K*_*d*_+[protein]). The mean *K*_*d*_ values are reported as mean ± SEM.

### Crystallization, data collection, and structure determination

The PPxY peptides and native WW domains were mixed (1:1 M ratio) at a final concentration of 10 to 30 mg/ml (20 mM Tris, pH 7.5, 150 mM NaCl). Crystals were initially screened using kits from Hampton Research. The cryoprotectant solution contained precipitant supplemented with 25 to 30% glycerol.

Apo-WW3 crystals were grown in a solution containing 3.6 M ammonium nitrate, 5% glycerol, and 0.1 M sodium acetate at pH 4.6 (20 °C). WW2–PPxY2 crystals were grown in a solution containing 2.0 M ammonium sulfate and 0.1 M Bis-Tris at pH 6.5 (20 °C). WW1–PPxY2 crystals were grown in a solution containing 1.6 M (NH_4_)_2_SO_4_, 0.1 M potassium sodium tartrate, 5% glycerol, and 0.1 M sodium acetate (37 °C).

Diffraction data were collected at SSRL beamline BL7-1 and processed using AutoXDS at SSRL http://smb.slac.stanford.edu/facilities/software/xds/#autoxds_script (40). Structures were determined by molecular replacement (Phaser in PHENIX (41)), using a solution structure of the NEDD4L WW3 domain (Protein Data Bank ID: 2MPT (31)) as a search model. Building was completed in Coot (42) and structures refined in PHENIX (43). Structure alignments, distance measurements, and figures were made using PyMol Molecular Graphics System (Schrödinger, LLC). Surface area accessibility was calculated using CoCoMaps (44).

### NMR data collection and structure refinement

#### NMR data collection and assignments

NMR samples were typically 1 mM of ^13^C/^15^N-labeled protein in the NMR buffer (20 mM sodium phosphate, 50 mM NaCl, and 10% ^2^H_2_O). All NMR spectra were collected at 25 °C on a Varian Inova 600 MHz spectrometer equipped with a triple-resonance ^1^H/^13^C/^15^N cryo-probe with z-axis pulsed field gradient capability. Backbone NMR chemical shift assignments were made using a suite of triple resonance experiments as described previously (45), using 3D versions of the HNCACB, CBCACONH, HNCO, and HA(CA)CO. Side-chain assignments were completed using 3D-H(CCO)NH-TOCSY, 3D-(H)C(CCO)NH-TOCSY, and 3D HCCH-TOCSY experiments. Aromatic resonances were assigned using a combination of ^1^H/^13^C HSQC, ^13^C-edited NOESY experiments centered on the aromatic carbon resonances (125 ppm), a ^13^C-edited NOESY experiment centered on the aliphatic region, and heteronuclear correlation experiments that correlate the Cβ carbons to the Hδ and Hε protons of the aromatic rings. Stereospecific assignments for β-methylene protons (and estimation of χ1 dihedral angles) were obtained using a combination of HNHB, HN(CO)HB, 15N-edited TOCSY, and NOESY data. Stereospecific assignments of side-chain methyl groups and qualitative determination of χ1 and χ2 dihedral propensities were obtained using long-range carbon–carbon and carbon–proton couplings observed in long-range carbon-carbon and long-range carbon-hydrogen couplings experiments. Three-dimensional ^15^N-edited NOESY-HSQC and 3D 13C-edited NOESY-HSQC’s (100 msec mixing times) were used to generate distance restraints for refinement (46, 47, 48). Dihedral restraints were derived from backbone Ha, Ca, CO, and Cb chemical shifts using TALOS. All spectra were processed with FELIX 2007 (Accelrys) or NMRPipe (49) and referenced indirectly to sodium trimethylsilylpropanesulfonate.

#### Structure determinations

Backbone and side-chain correlations were assigned, and nuclear Overhauser effect (NOE) intensities were integrated using tools in SPARKY https://www.cgl.ucsf.edu/home/sparky/ (50). The solution structure for WW3–PPxY1 and WW3 were refined using automated NOE assignments and torsion angle dynamics as implemented in CYANA (version 3.9) (51). Initial refinements used NOE data alone to define the overall fold. The final refinements added dihedral restraints, hydrogen bonds, and stereospecific assignments. A total of 100 randomized conformers were “folded” into 3D structures by iteratively including NOE constraints in a stepwise manner using criteria defined by CYANA. Of the 100 CYANA structures calculated, the 20 conformers with the lowest target function were subjected to a molecular dynamics protocol in the explicit solvent (52) using XPLOR-NIH (53).

Structures were validated using PROCHECK-NMR and AQUA and the programs supplied at the Protein Data Bank deposition site. All figures were created using PyMOL Molecular Graphics System (Schrödinger, LLC).

#### NMR chemical shift mapping

Chemical shift mapping of the interaction interface between the WW3 and PPxY1-3 was accomplished by making independent NMR samples of ^15^N-labeled WW3 (50 μM) supplemented with final concentrations of PPxY peptides over the range 0 to 150 μM in a titration NMR buffer (20 mM sodium phosphate [pH 6.0], 50 mM NaCl, and 10% ^2^H_2_O). ^1^H/^15^N -HSQC spectra were collected for each titration point. Normalized shift changes for ^1^H and ^15^N were calculated at the final titration point for each amide pair using the expression: Δn = [25∗((Δ^1^H)^2^+(Δ^15^N/5)^2^)]^0.5^.

## Data availability

Coordinates, structure factor, and NMR restraint and chemical shift files have been deposited in the Protein Data Bank (PDB) with the following codes: apo-WW3 (crystal, 7LP1), PPxY2-WW1 (7LP3), PPxY2-WW2 (7LP2), apo-WW3 (solution, 7LP4), and PPxY1-WW3 (7LP5).

## Supporting information

This article contains supporting information (54, 55, 56).

## Conflicts of interest

The authors declare that they have no conflicts of interest with the contents of this article.
