# Supplementary material for: Interactions between AMOT PPxY motifs and NEDD4L WW domains function in HIV-1 release
Source: J Biol Chem. 2021 Jul 17;297(2):100975. doi: 10.1016/j.jbc.2021.100975 (PMC8368996; doi:10.1016/j.jbc.2021.100975)
Supplement: Tables S1–S3 and Tables S1–S4 [file mmc1.pdf]

Interactions between AMOT PPxY Motifs and NEDD4L WW Domains Function in HIV-1 Release

Lara Rheinemann[1+], Tuscan Thompson[1+], Gaelle Mercenne[1], Elliott L. Paine[1], Francis C. Peterson[2], Brian F. Volkman[2], Steven L. Alam[1]*, Akram Alian[1]*, Wesley I. Sundquist[1]*

SUPPORTING INFORMATION TABLES AND FIGURES

Table S1. Data collection and refinement statistics

	**WW3 apo**	**WW2-PPxY2**	**WW1-PPxY2**
**PDB code**	7LP1	7LP2	7LP3
**Data collection**			
Space group	$P3_121$	$P42_12$	$P22_12_1$
Cell dimensions			
a, *b*, *c* (Å)	40.34, 40.34, 54.86	91.60, 91.60, 37.98	24.56, 55.52, 82.58
α,β,γ (°)	90.0, 90.0, 120.0	90.0, 90.0, 90.0	90.0, 90.0, 90.0
Resolution range (Å)	29.47 - 1.35	35.09 - 1.88	23.04 - 1.61
R_{meas}	0.0131 (0.2383)*	0.024 (0.35)	0.046 (0.39)
$I / \sigma I$	33.25 (4.26)	26.21 (2.82)	8.03 (2.53)
$CC_{1/2}$	1.0 (0.958)	1.0 (0.844)	1.0 (0.86)
Completeness (%)	99.83 (98.61)	99.95 (100)	99.56 (99.53)
No. unique reflections	11774 (1140)	13684 (1344)	15300 (1486)
Redundancy	2.0 (2.0)	2.0 (2.0)	2.0 (2.0)
**Refinement**			
R_{work} / R_{free}	0.184/0.213	0.185/0.228	0.178/0.21
Average *B*-factors (Å^2)	20.39	27.71	19.41
R.m.s. deviations			
Bond lengths (Å)	0.01	0.008	0.01
Bond angles (°)	1.12	0.96	1.12
Ramachandran (%)			
Favored	100	95.35	100
Allowed	0	4.65	0
Outliers	0	0	0
MolProbity score	1.09	1.86	1.15
No. TLS groups	5	13	7

*Numbers in parentheses correspond to the highest resolution shell

Table S2. NMR statistics

**Structure**		**WW3**	**WW3-PPxY1**
**PDB Code**		**7LP4**	**7LP5**
**Experimental constraints**			
Distance constraints for structure			
WW3		345	460
PPxY1		NA	134
Intermolecular		NA	64
Long		76	182
Medium [1<(i–j) ≤ 5]		43	72
Sequential [(i–j) = 1]		125	194
Intraresidue [i=j]		101	146
Total		345	594
Dihedral angle constraints (ϕ and ψ)		68	84
Number of restrains per residue		9	11.3
Number of long-range restrains per residue		1.7	3.0
**Average atomic R.M.S.D. to the mean structure (Å)**			
Residues		498-526	102-113,498-529
Backbone (C^{α}, C′, N)		0.50 ± 0.12	0.51 ± 0.09
Heavy atoms		0.94 ± 0.12	0.99 ± 0.10
**Deviations from idealized covalent geometry**			
Bond lengths	RMSD (Å)	0.014	0.014
Torsion angle violations	RMSD (°)	1.1	1.2
**Constraint violations**			
NOE distance	Number > 0.5 Å[a]	0 ± 0	0 ± 0
NOE distance	RMSD (Å)	0.023 ± 0.002	0.020 ± 0.002
Torsion-angle violations	Number > 5 °[b]	0.0 ± 0	0.0 ± 0
Torsion-angle violations	RMSD (°)	0.549 ± 0.11	0.716 ± 0.60
**Global quality scores (raw/Z score)**[c]			
Verify3D		0.03	0.23
Prosall		0.11	0.25
PROCHECK (ϕ–ψ)[d]		-0.57	-0.41
PROCHECK (all)[d]		-0.42	-0.42
MolProbity clash score		8.31	12.57
**Ramachandran statistics (% of all residues)**[e]			
Most favored		89.8	88.6
Additionally allowed		10.0	11.4
Generously allowed		0.2	0
Disallowed		0	0

[a] The largest NOE violation in the ensemble of structures for WW3 and WW3-PPxY1 were 0.30 Å and 0.55 Å, respectively. [b] The largest torsion-angle violation in the ensemble of structures for WW3 and WW3-PPxY1 were 4.80° and 5.1°, respectively. [c] Calculated using PSVS version 1.5 [d] Based on ordered residues 498-535 for WW3 and ordered residues 105-114 and 498-532 for WW3-PPxY1. [e]Ramachandran statistics calculated using PROCHECK for ordered residues 498-535 for WW3 and ordered residues 105-114 and 498-532 for WW3-PPxY1.

Table S3. Plasmids used in this study.

Plasmid name	Source	Plasmid repository
pGEX-PP-NEDD4L-WW1(193-226)	Generated for this study	Addgene, #166701
pGEX-PP-NEDD4L-WW2(385-418)	Generated for this study	Addgene, #166702
pGEX-PP-NEDD4L-WW3(497-530)	Generated for this study	Addgene, #166703
pGEX-PP-NEDD4L-WW4(548-581)	Generated for this study	Addgene, #166704
pCA528-PPxY1-GS-WW3e	Generated for this study	Addgene, #166705
pET29-sortase A pentamutant	Kind gift from David Liu	Addgene, #75144
pCA528-AMOT(1-300)-sortase	Generated for this study	Addgene, #166706
HIV-1_{NL4-3} $R9_{\Delta PTAP,\Delta YP}$	(54)	
pCI-Flag-NEDD4L	(19)	DNASU HsCD00671550
pCI-Flag-NEDD4LΔWW	(19)	DNASU HsCD00671555
pCI-Flag-NEDD4LΔWW1	Generated for this study	Addgene, #166707
pCI-Flag-NEDD4LΔWW2	Generated for this study	Addgene, #166708
pCI-Flag-NEDD4LΔWW3	Generated for this study	Addgene, #166709
pCI-Flag-NEDD4LΔWW4	Generated for this study	Addgene, #166710
pCI-Flag-NEDD4LΔWW1,2,4	Generated for this study	Addgene, #171137
pcDNA3HA-AMOT p130	(19)	DNASU HsCD00671559
pcDNA3HA-AMOT p130ΔPPxY	(19)	DNASU HsCD00671563
pcDNA3HA-AMOT p130ΔPPxY1	Generated for this study	Addgene, #166711
pcDNA3HA-AMOT p130ΔPPxY2	Generated for this study	Addgene, #166712
pcDNA3HA-AMOT p130ΔPPxY3	Generated for this study	Addgene, #166713
pcDNA3HA-AMOT p130ΔPPxY1(L106P)	Generated for this study	Addgene, #166714
pcDNA3HA-AMOT p130ΔPPxY1(A112K)	Generated for this study	Addgene, #166715
pcDNA3HA-AMOT p130ΔPPxY1(E104K/E105K)	Generated for this study	Addgene, #171130

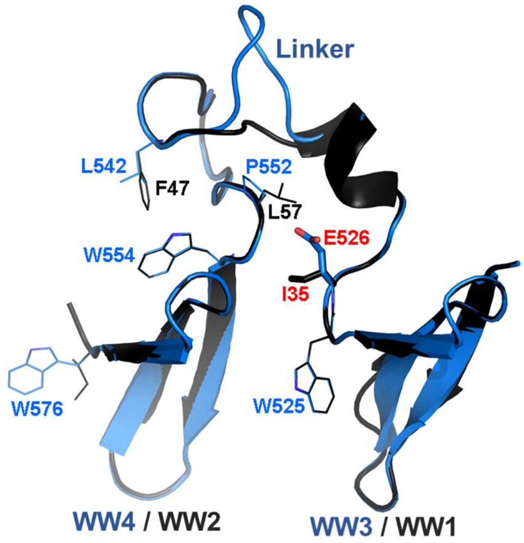

Figure S1. Structure of the KIBRA WW1-WW2 tandem domain (PDB: 6J68) (black) and model of the (hypothetical) NEDD4L WW3-WW4 domain-tandem in the same conformation (blue). The NEDD4L WW3-WW4 tandem domains were modeled using SwissModel (55). Note that the WW interdomain interface is expected to be destabilized by substitution of residue Ile^{35} in the KIBRA WW1-WW2 structure by Glu^{526} in NEDD4L WW3-WW4 (shown in sticks and labeled red, see text for details).

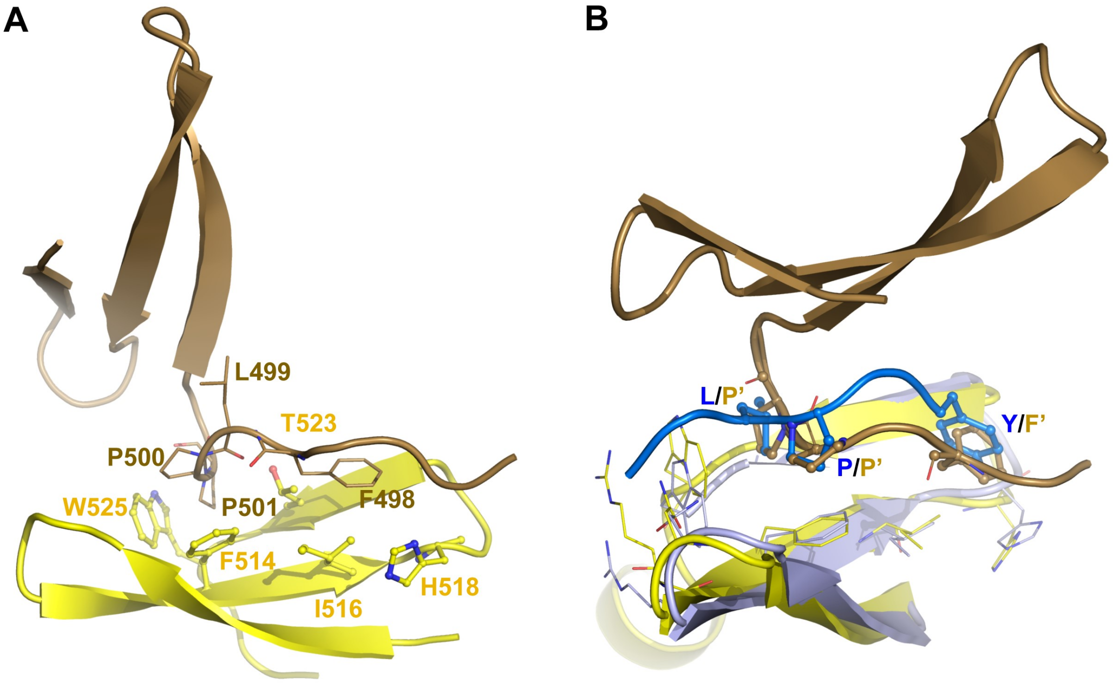

Figure S2. Crystal structure of the NEDD4L WW3 domain. *A,* structure of WW3 domain showing how the N-terminal $_{498}$FLPP$_{501}$ tail of one WW domain (brown) binds a symmetry mate (yellow) in the crystal lattice. The FLPP tail occupies the core peptide binding site and contacts residues Phe^{514}, Ile^{516}, His^{518}, Trp^{525}, and Thr^{523}, but adopts the opposite orientation as that seen for PPxY peptides. *B,* structure of WW3 domain (pale blue) bound to AMOT PPxY1 (blue, LPxY) is superimposed to WW3 apo (yellow) bound to a cryptic peptide (brown, FxPP) from WW3 symmetry mate as in *A*. Cryptic peptide binds in a flipped orientation (P´P´xF´).

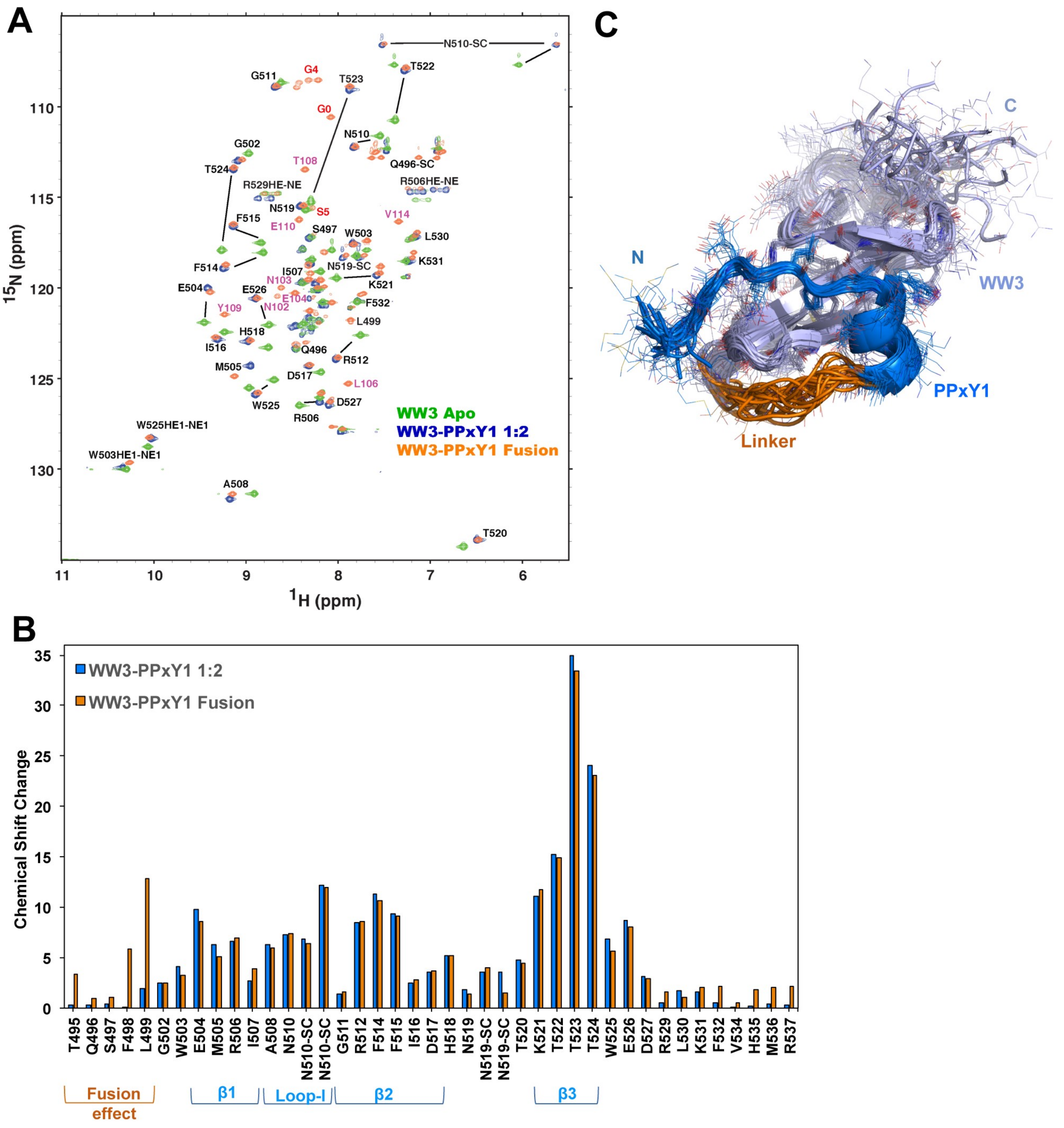

Figure S3. The fused AMOT PPxY1 binds NEDD4L WW3 in a native conformation. *A,* overlay of ^{1}H/^{15}N HSQC spectra of ^{15}N-labeled WW3-PPxY1 fusion construct (orange) with apo ^{15}N-labeled WW3 (green) and ^{15}N-labeled WW3 in the presence of two equivalents of PPxY1 peptide (blue). Note that selected residues shift dramatically upon peptide binding (labeled) and that the chemical shifts of these residues overlay almost perfectly with the equivalent residues in the WW3-PPxY1 fusion construct.

B, pairwise chemical shift changes (from *A*) of WW3-PPxY1 fusion (orange) and WW3 in the presence of two equivalents of PPxY1 (blue). Characteristic WW-domain secondary structure elements (shown in *C*) are indicated. Normalized shift changes for ^{1}H and ^{15}N were calculated at the final titration point for each amide pair using the expression: $\Delta n=[25*((\Delta^{1}H)^{2}+((\Delta^{15}N))/5)^{2})]^{0.5}$.

C, overlay of the 20 lowest energy structures of the WW3-PPxY1 fusion protein, showing that the PPxY1 (blue), linker (orange) and WW3 domain (light blue) elements are well ordered, except at the WW3 domain C-terminus.

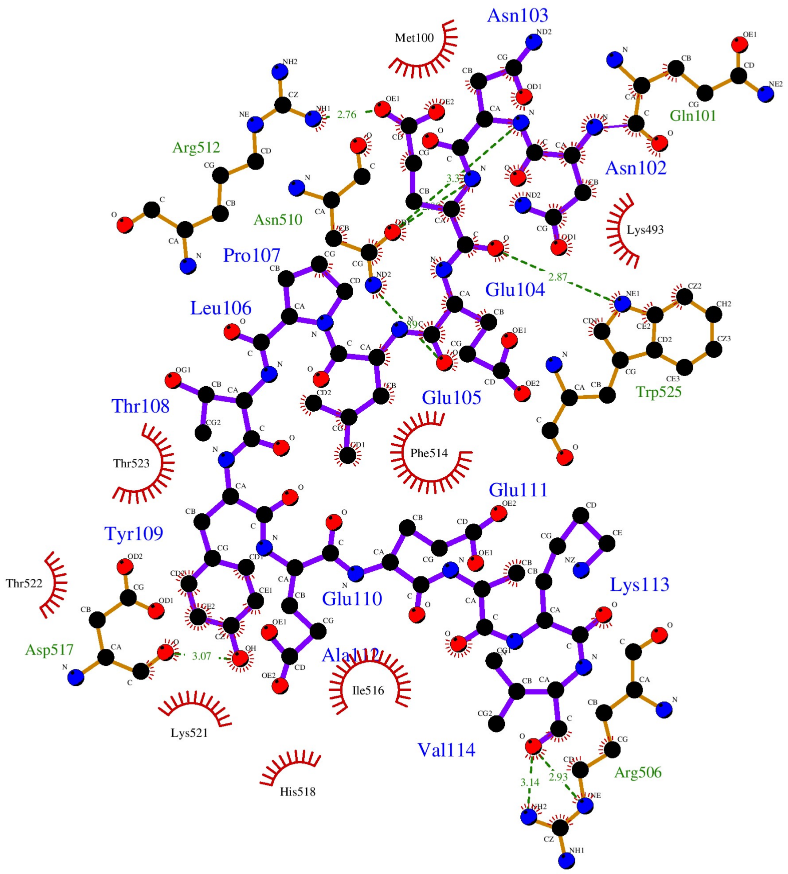

Figure S4. Detailed interactions of WW3 and PPxY1. PPxY1 in purple (blue labels), WW3 in brown (green labels). Hydrogen bonds are shown with dashed green lines and green labels for corresponding distances. Hydrophobic interactions are shown with red spikes and black labels for WW3 residues. Spheres represent atoms of carbon (black), nitrogen (blue) and oxygen (red). Figure generated using LigPlot+ (56) and the lowest energy structure. Other ensemble structures will result in different interactions.
